# A large number of cerebral microbleeds in CADASIL patients presenting with recurrent seizures: a case report

**DOI:** 10.1186/s12883-019-1342-2

**Published:** 2019-05-30

**Authors:** Chumpol Anamnart, Dittapong Songsaeng, Sirisak Chanprasert

**Affiliations:** 1 Division of Neurology, Department of Medicine, Prapokklao Hospital, Faculty of Medicine, Chulalongkorn University, 38 Leab Noen Rd, Tambon Wat Mai, Mueang District, Chantaburi Province 22000 Thailand; 2 Department of Radiology, Siriraj Hospital, Faculty of Medicine, Mahidol University, 2 Prannok Rd, Bangkoknoi, Bangkok 10700 Thailand; 3 Division of Medical Genetics, Department of Medicine, University of Washington, 1959 NE Pacific St, Seattle, WA 981957720 USA

**Keywords:** CADASIL, Cerebral microbleeds, *NOTCH3* gene, Homozygous p. Arg558Cys, Case report

## Abstract

**Background:**

Cerebral autosomal dominant arteriopathy with subcortical infarcts and leukoencephalopathy (CADASIL) is a hereditary arteriopathy associated with the *NOTCH3* gene. Clinical manifestations include strokes, transient ischaemic events, psychiatric disturbances, dementia, and migraines. We report a case of a Thai man with a severe CADASIL phenotype who presented with recurrent seizures and acute ischaemic stroke and classic vascular risk factors.

**Case presentation:**

A 50-year-old man with a history of mood disorder and progressive cognitive decline for 20 years as well as well-controlled diabetes mellitus and hypertension presented with recurrent generalized seizures and acute right-sided weakness. An MRI of the brain showed acute infarction of the left pons, a large number of cerebral microbleeds throughout the brain and white matter abnormalities without classic anterior temporal lobe lesions. Molecular genetic testing identified a homozygous pathologic variant, c.1672C > T (p. Arg558Cys), in the *NOTCH3* gene. The diagnosis of CADASIL was confirmed. His clinical symptoms deteriorated, and he died of tracheobronchitis with secretion obstruction.

**Conclusion:**

This case raises awareness of an uncommon cause of acute ischaemic stroke in patients with classic vascular risk factors and emphasizes the need for a complete evaluation in cases with unexpected clinical presentation or unexpected diagnostic study results.

## Background

Cerebral autosomal dominant arteriopathy with subcortical infarcts and leukoencephalopathy (CADASIL) is the most frequent genetic cause of stroke [1]. Clinical manifestations include strokes, transient ischaemic events, psychiatric disturbances, dementia, and migraines [2]. The classic clinical presentation is a young or middle-aged adult experiencing ischaemic stroke or transient ischaemic attack and/or early onset dementia with subcortical leukoencephalopathy on brain imaging [2]. Seizures are an uncommon symptom found in only 6 to 10% of cases [2–4]. The exact aetiology of epileptic seizures is not known. The common MRI findings are T2-hyperintense lesions in the periventricular and subcortical white matter, especially the external capsule and anterior temporal lobes, which are more likely to be involved in this condition than in sporadic forms of small vessel diseases [5]. The prevalence of cerebral microbleeds ranges from 34 to 75% [3, 4, 6–8]. Common locations of cerebral microbleeds are in the thalamus, basal ganglia, subcortical white matter, brainstem, cerebellum and grey-white matter junction [7, 8]. A diagnosis of CADASIL requires the identification of genetic changes in the *NOTCH3* gene, which is located on chromosome 19 [1]. Here, we report a Thai man with a previous history of well-controlled diabetes mellitus and hypertension who presented with seizures and acute right-sided weakness. An MRI of the brain showed acute infarction in the left pons and numerous cerebral microbleeds throughout the brain.

## Case presentation

**Fig. 1:**
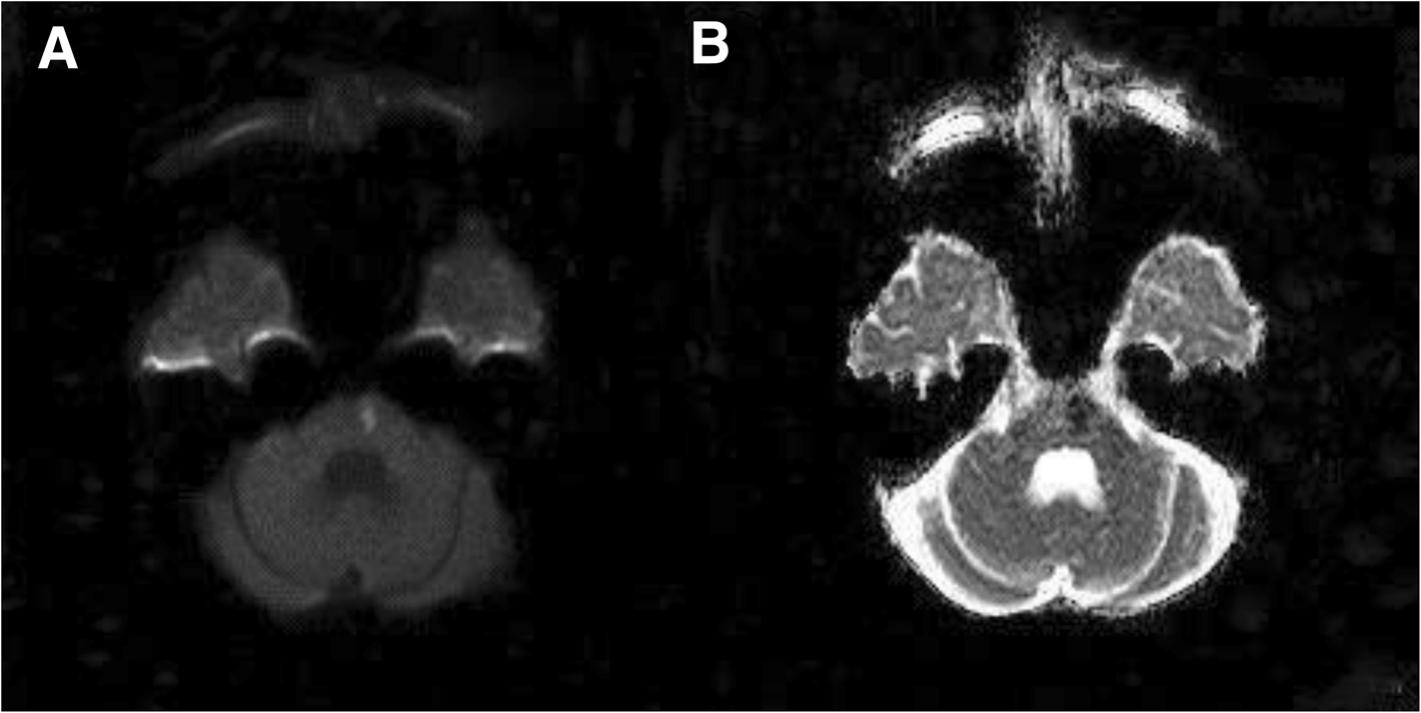
Brain MRI of the patient. Diffusion weighted imaging (**a**) and apparent diffusion coefficient image (**b**) showing acute infarction in the left anterior paramedian of the mid-pons

**Fig. 2:**
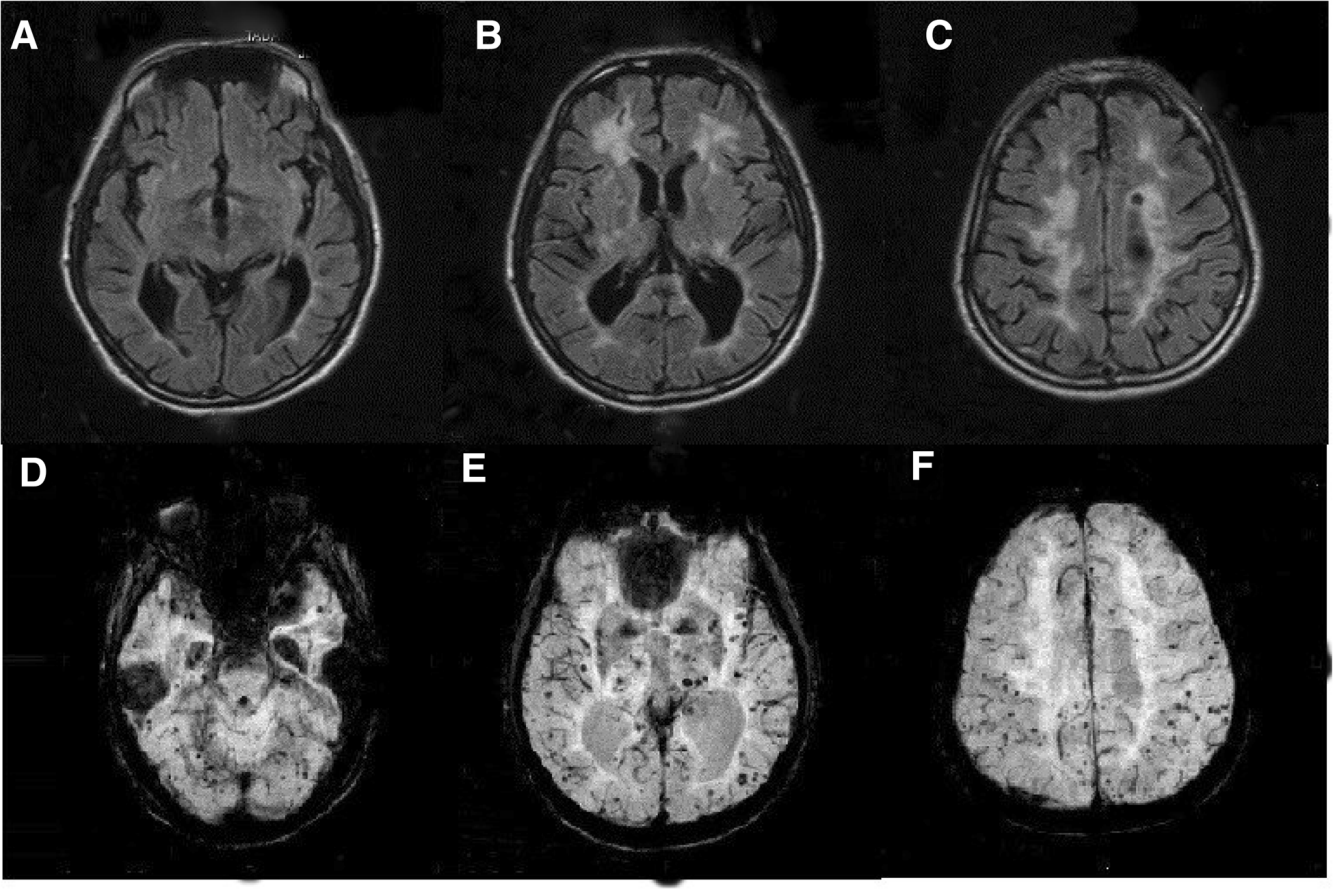
Brain MRI of the patient. T2-weighted fluid-attenuated inversion recovery (**a**, **b**, **c**) showing patchy periventricular white matter changes, predominantly at the bilateral frontal horns of the lateral ventricles, bilateral centrum semiovales with small old infarctions at the anterior right frontal subcortical white matter and left frontal periventricular white matter, and susceptibility weighted images (**d**, **e**, **f**) showing numerous microbleeds scattered throughout the bilateral cerebral hemispheres, cerebellum, bilateral basal ganglia and thalamus

A 50-year-old Thai male presented with three episodes of generalized seizures and right-sided hemiparesis for 6 h before arrival. He had no previous seizures. He had a history of well-controlled diabetes mellitus and hypertension for 20 years and took metformin 1000 mg/day and diltiazem 60 mg/day. His past medical history revealed progressive slowness in thinking and walking, memory impairment, sleep-wake disturbance and mood disorder, which had slowly progressed for the past 20 years; however, it had rapidly worsened during last year of his life. He was diagnosed with organic mood disorder 5 years before this presentation and treated with risperidone 0.5 mg/day, sertraline 50 mg/day, and trihexyphenidyl 1 mg/day. Even with this treatment, his symptoms had been progressively worsening for the past 1 year to the point that he could not perform daily living activities, such as taking the correct medications. He was the fifth of seven children. His sister had a history of unexplained hearing loss, cognitive decline, and slowness of movement starting at the age of 20. When she was 40 years old, she developed visual and auditory hallucinations as well as recurrent transient ischaemic attacks with full recovery. The patient’s father and mother died at the ages of 70 and 78, respectively, and had no history of cognitive impairment or stroke. A mental status examination showed a good level of consciousness; however, he was mute and slow to respond to commands. A motor examination showed right-sided weakness (grade 2/5 for arm and grade 0/5 for leg) and generalized hyperreflexia except for right leg hyporeflexia and no sensory impairment. There was also mild right facial weakness. A CT of the brain showed diffuse white matter abnormalities, old multiple lacunar infarctions in the bilateral basal ganglia, thalamus, and left pons. The initial diagnosis was acute ischaemic stroke with seizures. He was prescribed 300 mg/day of aspirin and usual stroke care. Phenytoin was prescribed for seizure control. An MRI of the brain was performed on day 12 after admission. The results showed acute infarction of the left pons (Fig. 1), several old lacunar infarcts surrounded by minimal gliosis in the bilateral putamen and thalamus as well as few scattered small, old infarcts surrounded by minimal gliosis in the bilateral frontal-parietal periventricular white matter without anterior temporal lobe lesion (Fig. 2 a, b, and c). Surprisingly, a large number of microbleeds were found throughout the brain (Fig. 2 d, e and f). The total numbers of cerebral microbleeds was 214 and 136 in lobar areas according to the Microbleeds Anatomical Rating Scale (MARS) [9]. After a comprehensive review of vascular risk factors, his HbA1C was 5.5%, and his serum LDL was 79 mg/dL. His blood pressure was well-controlled. None of these risk factors explained his symptoms and MRI findings. He had a history of unexplained cognitive impairment and mood disorder. In addition, his sister had a history of cognitive decline, psychiatric symptoms and transient ischaemic attack; therefore, a genetic condition, such as CADASIL, was suspected and confirmed by molecular genetic testing, which revealed a homozygous known pathologic variant, c.1672C > T (p. Arg558Cys), in the *NOTCH3* gene. His clinical symptoms deteriorated, and he died of tracheobronchitis with secretion obstruction.

## Discussion and conclusions

We report a patient with a history of unexplained progressive cognitive impairment and mood disorder who presented to our institution with recurrent seizures and acute ischaemic stroke. Brain MRI findings demonstrated a large number of cerebral microbleeds that did not correlate with his vascular risk factors. In addition, his sister had a history of cognitive impairment, psychiatric symptoms and transient ischaemic attack. A genetic study revealed a homozygous known pathologic variant in the *NOTCH3* gene.

Cerebral microbleeds represent perivascular hemosiderin depositions and are observed in several conditions, including patients with hypertensive arteriopathy, cerebral amyloid angiopathy and CADASIL. Cerebral microbleeds are important markers of the structural integrity of small blood vessels [10]. In CADASIL, the prevalence of cerebral microbleeds ranges from 34 to 75% [3, 4, 6–8]. Common locations of cerebral microbleeds are the thalamus, the basal ganglia, the subcortical white matter, the brainstem, the cerebellum and the grey-white matter junction [7, 8]. In this patient, however, the cerebral microbleeds were diffusely distributed throughout the brain. To the best of our knowledge, this case presented with the largest number of cerebral microbleeds ever recorded. A previous study showed that in CADASIL, the number of cerebral microbleeds is correlated with age [8]. One study in particular reported a higher total number of cerebral microbleeds was associated with haemorrhagic stroke, dementia, and urge incontinence after adjusting for age [4].

Previous reports have demonstrated that cerebral microbleeds are associated with poor functional outcomes and can help identify a severe form of CADASIL [6]. While the exact underlying pathophysiology of CADASIL has yet to be identified, recent studies have shown endothelial dysfunction and impaired smooth muscle cell relaxation occur in small cerebral blood vessels. We suggest that the large number of cerebral microbleeds observed in this case were caused by a homozygous state and potentially uncontrolled diabetes and hypertension before he was followed up at our hospital. This pathogenic variant, p. Arg558Cys, was previously found in a homozygous state in only two Portuguese patients with CADASIL. One of those patients had a more severe and early phenotype [11]. Among the other aetiologies of cerebral microbleeds, cerebral amyloid angiopathy should also be considered, but in this case, the patient showed no evidence of lobar haemorrhage. In contrast to CADASIL, in amyloid angiopathy, cerebral microbleeds usually spare the deep grey matter and brainstem**.**

Seizures are an uncommon symptom found in approximately 6 to 10% of patients with CADASIL [2–4]. The underlying pathophysiology of seizure in this case has yet to be defined. Nonetheless, evidence suggests that white matter lesions near the cortex are epileptogenic [12, 13]. Hence, we believe that our patient developed seizures because he had a large number of cerebral mibrobleeds in cortical regions.

In summary, we present a case of CADASIL to raise awareness of this rare and devastating condition. We demonstrate that in patients with classic vascular risk factors, acute ischaemic stroke can be caused by other uncommon conditions. We must therefore think more broadly, especially in patients with unexpected clinical presentations or diagnostic study results, such as the seizures and large number of cerebral microbleeds observed in this patient. Finally, we investigated the aetiology of mood disorders and cognitive impairment, especially with early onset presentation.

## Data Availability

Not applicable.

## Abbreviations

CADASIL: Cerebral autosomal dominant arteriopathy with subcortical infarcts and leukoencephalopathy
CT: Computed tomography
HbA1C: Haemoglobin A1C
LDL: Low-density lipoprotein
MRI: Magnetic resonance imaging

## References

[1] Joutel A, Corpechot C, Ducros A, Vahedi K, Chabriat H, Mouton P (1996). Notch3 mutations in CADASIL: a hereditary adult-onset condition causing stroke and dementia. Nature..

[2] Dichgans M, Mayer M, Uttner I, Brüning R, Müller-Höcker J, Rungger G (1998). The phenotypic spectrum of CADASIL: clinical findings in 102 cases. Ann Neurol.

[3] Bersano A, Bedini G, Markus HS, Vitali P, Tibaldi EC, Taroni F (2018). The role of clinical and neuroimaging features in the diagnosis of CADASIL. J Neurol.

[4] Nannucci S, Rinnoci V, Pracucci G, MacKinnon AD, Pescini F, Adib-Samii P (2018). Location, number and factors associated with cerebral microbleeds in an Italian-British cohort of CADASIL patients. PLoS ONE.

[5] O’Sullivan M, Jarosz JM, Martin RJ, Deasy N, Powell JF, Markus HS (2001). MRI hyperintensities of the temporal lobe and external capsule in patients with CADASIL. Neurology..

[6] Puy L, De Guio F, Godin O, Duering M, Dichgans M, Chabriat H (2017). Cerebral microbleeds and the risk of incident ischemic stroke in CADASIL (cerebral autosomal dominant arteriopathy with subcortical infarcts and leukoencephalopathy). Stroke..

[7] Lee JS, Ko KH, Oh JH, Park JH, Lee HK, Floriolli D, et al. Cerebral microbleeds, hypertension, and intracerebral hemorrhage in cerebral autosomal-dominant arteriopathy with subcortical infarcts and leukoencephalopathy. Front Neurol. 8(MAY) 10.3389/fneur.2017.00203. 10.3389/fneur.2017.00203 PMC5430055 28555127

[8] Dichgans M, Holtmannspötter M, Herzog J, Peters N, Bergmann M, Yousry TA (2002). Cerebral microbleeds in CADASIL: a gradient-echo magnetic resonance imaging and autopsy study. Stroke..

[9] Gregoire SM, Chaudhary UJ, Brown MM, Yousry TA, Kallis C, Jäger HR, Werring (2009). The Microbleed Anatomical Rating Scale (MARS): reliability of a tool to map brain microbleeds. Neurology..

[10] Viswanathan A, Chabriat H (2006). Cerebral microhemorrhage. Stroke..

[11] Lopes-de-Almeida M, Ramos L, Cordeiro G, Almeida R, Sa J, Saraiva JM. A portuguese family with CADASIL diagnosis with anticipation age of onset observed. Poster presented at 44th Genetics Conference Doutor Jacinto Magalhães; 2015; Porto, Portugal.

[12] Oh JH, Kang BS, Choi JC (2016). CADASIL initially presented with a seizure. J Epilepsy Res.

[13] Velizarova R, Mourand I, Serafini A, Crespel A, Gelisse P (2001). Focal epilepsy as first symptoms in CADASIL. Seizure..

